# The shape of the corpus callosum is different in bipolar disorder

**DOI:** 10.1017/neu.2024.51

**Published:** 2024-11-06

**Authors:** Mustafa Nuray Namli, Sema Baykara, Ozlem Gul, Murat Baykara

**Affiliations:** 1 Department of Psychiatry, Bakirkoy Research and Training Hospital for Psychiatric and Neurological Diseases, Bakirkoy, Istanbul, Turkiye; 2 Department of Psychiatry, Erenkoy Psychiatry and Neurology Training and Research Hospital, Istanbul, Turkiye; 3 Department of Psychiatry, Istinye University, Istanbul, Turkiye; 4 Department of Radiology, Haydarpasa Numune Training and Research Hospital, Istanbul, Turkiye

**Keywords:** Corpus callosum, bipolar disorder, magnetic resonance imaging, neuroimaging, computer-assisted image processing

## Abstract

**Objective:**

Bipolar disorder (BD) is a recurrent chronic disorder characterised by fluctuations in mood and energy disposition. Diseases could lead to degenerative alterations in brain structures such as corpus callosum (CC). Studies demonstrated that abnormalities in CC are associated with BD symptoms. The present study aims to analyse the CC of the patients with statistical shape analysis (SSA) and compare the findings with healthy controls.

**Methods:**

Forty-one BD patients and 41 healthy individuals in similar age groups, which included 23 female and 18 male subjects, participated in the study. CC was marked with landmarks on the mid-sagittal images of each individual. The mean ‘Procrustes’ point was calculated, and shape deformations were analysed with thin-plate spline analysis.

**Results:**

Significant differences were observed in the shape of CC between the two groups, where maximum CC deformation was observed in posterior region marks in BD patients. There was no significant difference between the CC area of the BD patients and controls.

**Conclusions:**

CC analysis conducted with SSA revealed significant differences between patients and healthy controls. The study findings emphasised the abnormal distribution of white matter in CC and the variable subregional nature of CC in BD patients. This study may enable the development of more targeted and effective treatment strategies by taking into account biological factors and understanding the differences in the brain regions of individuals with BD.

Significant outcomes

- Significant shape differences were seen between MRI images of BD patients and healthy controls.
- There was no significant difference between the CC area of BD patients and controls.
- Patients with BD had an abnormal distribution of white matter in the CC and variable subregional structure of the CC.

Limitations

- The small patient and control sample size were limitations of the current study.
- A scale was not applied to determine the clinical characteristics of the patients.
- The patients evaluated in the study receive antipsychotic and mood stabiliser medication.

## Introduction

Bipolar disorder (BD) is a continuous chronic disorder characterised by mood and energy variations. It affects more than 1% of the global population, independent of nationality, ethnicity, or socio-economic status. BD is among the main causes of disability in youth, leading to cognitive and functional impairment and high mortality, especially by suicide (Rowland and Marwaha, 2018).

Bipolar (affective) disorder, originally called manic depressive illness, is a multifactorial disorder of unknown etiology and is one of the most difficult psychiatric disorders to treat. It is important to be aware of potential risk factors so that clinicians can identify cases that are more likely to develop BD. Ideally, identifying the factors that directly lead to BD would enable interventions to be made at the individual or societal level. This will help prevent the development of the disease and improve outcomes through early treatment (Rowland and Marwaha, 2018).

The corpus callosum (CC) connects the cortical regions in both hemispheres and is the largest white matter (WM) structure in the human brain. Studies on complete and partial callosotomy or callosal lesions have provided further data on the functions of CC, demonstrating that it facilitates communication between cerebral hemispheres (van der Knaap and van der Ham, 2011). Furthermore, CC agenesis is rare, and associated neuropsychiatric disorders include epilepsy, Asperger’s syndrome, learning problems, depression, schizophrenia, conduct disorders, and conversion symptoms (Bhatia *et al*., 2016).

The CC plays a key role in central nervous system diseases. CC volume correlates with the severity and/or extent of neurodegenerative diseases. Although the role of CC has been extensively studied over the last decade and different CC segmentation and parcellation algorithms and methods have been published, no reviews or surveys have been published on these developments (Cover *et al*., 2018). CC has been associated with cognitive and emotional deficits in several neuropsychiatric and mood disorders (Cyprien *et al*., 2011).

Quantitative morphologic analysis of individual brain structures with neuroimaging frequently involves segmentations determined by volumetric measurements. Volumetric alterations could serve as intuitive markers since these might explain disease-induced atrophies or enlargement of structures. On the other hand, structural alterations such as bending/flattening or changes in a specific part of a structure, for example, thickening of the occipital horn of ventricles, are not adequately reflected in global volumetric measurements. The development of new methods to analyse three-dimensional shapes requires the development of mathematical methods for 2D analyses on statistical biological variability to address this issue (Styner *et al*., 2003).

This study is a structural neuroimaging study of CC with the SSA approach on a group of patients diagnosed with BD. The main aim was to determine whether, to what extent, the disorder is associated with structural changes in the CC. CC was assessed by computational analysis of T2-weighted MRI scans of BD patients and compared these findings with those of healthy controls.

## Material and methods

### Participants and study design

The present cross-sectional retrospective study was conducted with the data of the patients who met the Diagnostic and Statistical Manual of Mental Disorders-5 (DSM-5) criteria (American Psychiatric Association, 2013). for BD, admitted to the outpatient unit or the inpatient clinic at a Psychiatric Training and Research Hospital between January 2018 and July 2022. The Local Ethics Committee approved the study (IRB: 07/07/2022–2022.07.175). The cases diagnosed with BD based on the DSM-5 criteria met the inclusion criteria and underwent cranial MRI examination were determined. And the images of 41 cases identified in the Hospital Information System (HIS) were analysed by a radiologist, and the radiologist determined the suitability of the case for the study. Inclusion criteria, as determined by the HIS data and patient statements, included age between 18 and 65, no concomitant psychiatric diagnosis such as mental retardation or neurological or physiological disease, and no history of alcohol or substance abuse during the last 6 months. The control group included age- and gender-matched healthy individuals who met the above-mentioned study criteria without any psychiatric diagnosis and underwent brain MRI examination for other reasons.

### Data collection and image analysis

All participants were scanned by a 1.5T Philips Ingenia MRI scanner (Philips Medical System, Best, NL) with an 8-channel array head coil. T2 Turbo Spin Echo MRI images were obtained.

### Two-dimensional landmarks

**Figure 1.**
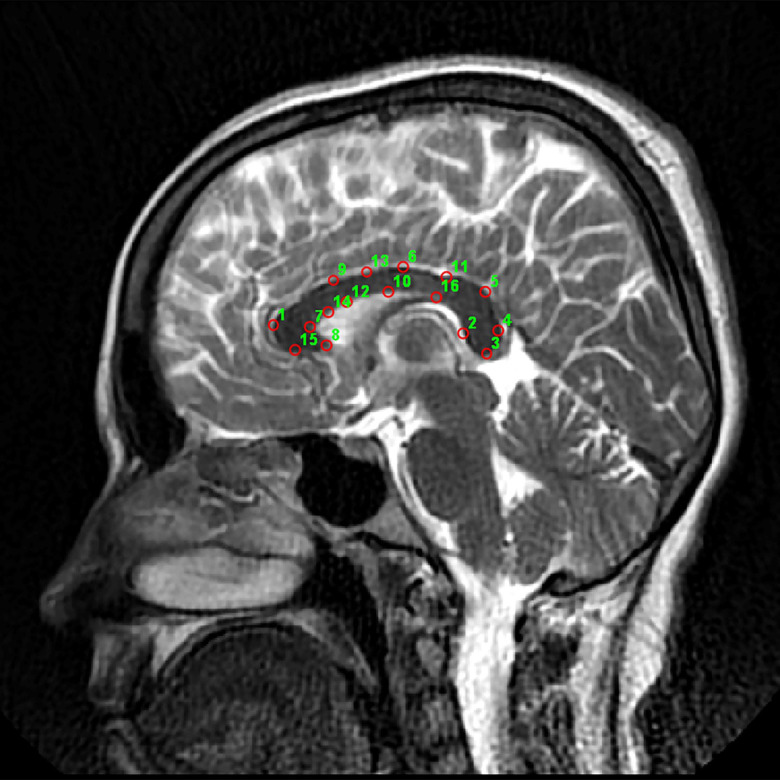
‘Procrustes’ landmark points in a midline sagittal image.

Mid-sagittal T2-weighted two-dimensional digital MRI images that most clearly reflected the cerebral aqueduct, CC, and superior colliculus were selected for each individual (Fig. 1). CC was marked (Fig. 1) with TpsDig2 version 2.32 software on each selected image with standardised anatomical landmarks (Sampson *et al*., 2001; Ozdemir *et al*., 2007; Baykara *et al*., 2022), completing the data collection procedure (Hystrix, 2015).

### Statistical deformation analysis

The mean ‘Procrustes’ landmark point was calculated, and shape deformations were analysed with the thin-plate spline (TPS) analysis with Past version 4.10 software (Hammer *et al*., 2001). Areas with the highest expansion or contraction were marked with different colours to indicate deformations in the analysis. The homogeneity of the variance-covariance matrices was determined with the Box-M test (Hammer *et al*., 2001; Dryden and Mardia, 2016). Due to the non-homogeneity of the matrices, the resampling-based James Fᴊ test was employed to compare CC shapes in the control and BD groups (Dryden and Mardia, 2016; Team, T.R.C., R. 2021, The R Foundation, p. Free Software Foundation’s GNU General Public License). Furthermore, the root mean squares of Kendall’s Riemann distance (rho) were compared with the mean shape to determine overall shape variations in the control and BD groups. The allometric analysis was conducted with the multivariate regression analysis of centroid sizes (CS, the square root of the sum of the square of the Euclidean distances between each sign and the centre) and tangent coordinates. Model significance was determined with the Wilks’ lambda test. The fitness of the model was determined by the mean square error (MSE) and the determination coefficient (R^2^) (He *et al*., 2010; Kaya *et al*., 2019; Sigirli *et al*., 2020).

### Landmark reliability

In the present study, a single rater manually determined all landmarks. Intra-rater reliability was not calculated since previous studies reported high rater reliability in landmark selection (Kaya *et al*., 2019; Sigirli *et al*., 2020).

### Statistical analysis

Shapes version 1.2.6, R version 4.2.0, and PAST version 4.10 software were employed in the statistical shape analysis (SSA) (Hammer *et al*., 2001; Dryden and Mardia, 2016). Statistical analyses were conducted on IBM statistics software (SPSS for Windows version 26, IBM Corporation, Armonk, New York, USA). A p-value of less than 0.05 was considered statistically significant.

## Results

Eighteen of the 41 patients and 18 of the 41 control subjects were male, and there was no significant difference between the groups based on gender. The mean age was 34.17 ± 10.14 in the patient group and 35.10 ± 9.92 in the control group, and there was no significant difference between the groups based on age (*p* = 0.676).

**Figure 2.**
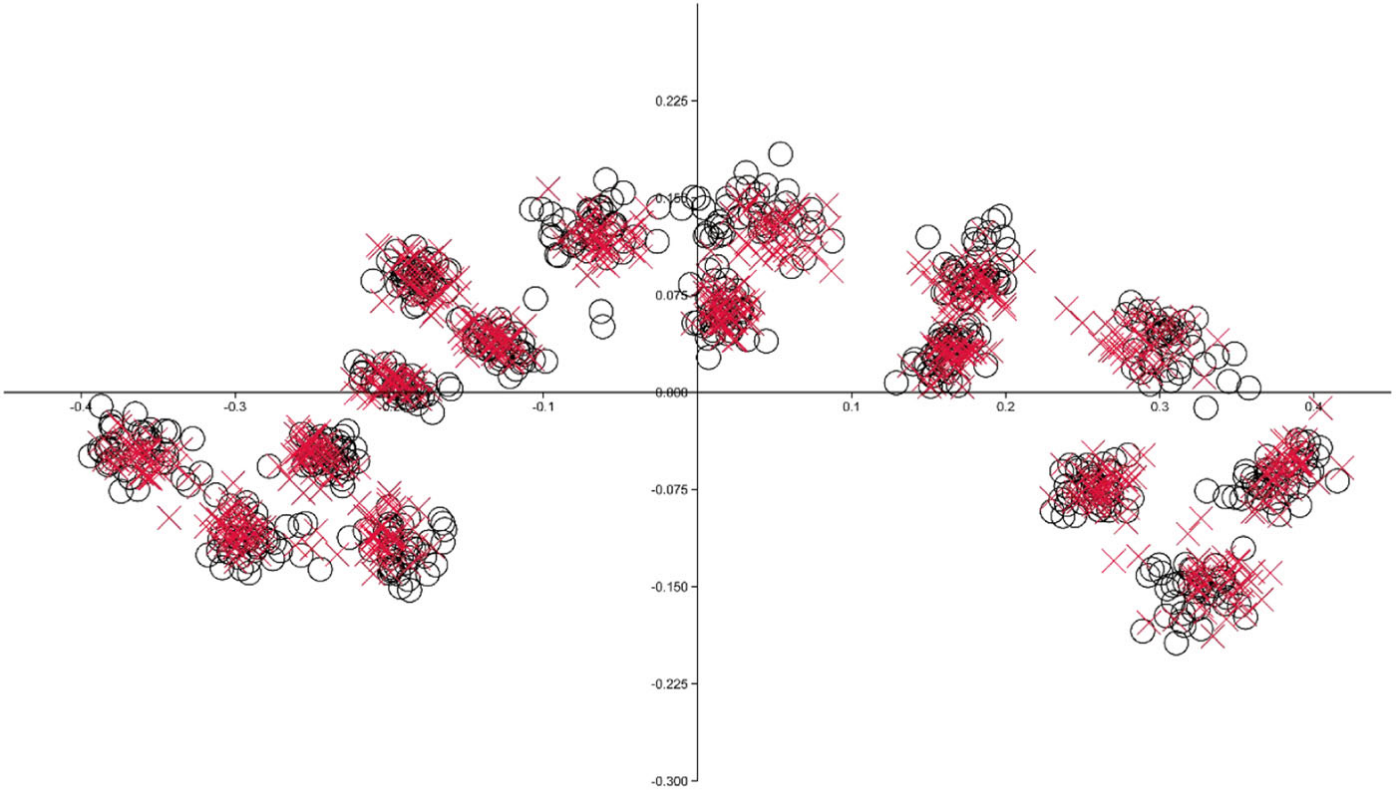
Landmark scatter plot for controls (O) and bipolar disorder patients (X).

**Figure 3.**
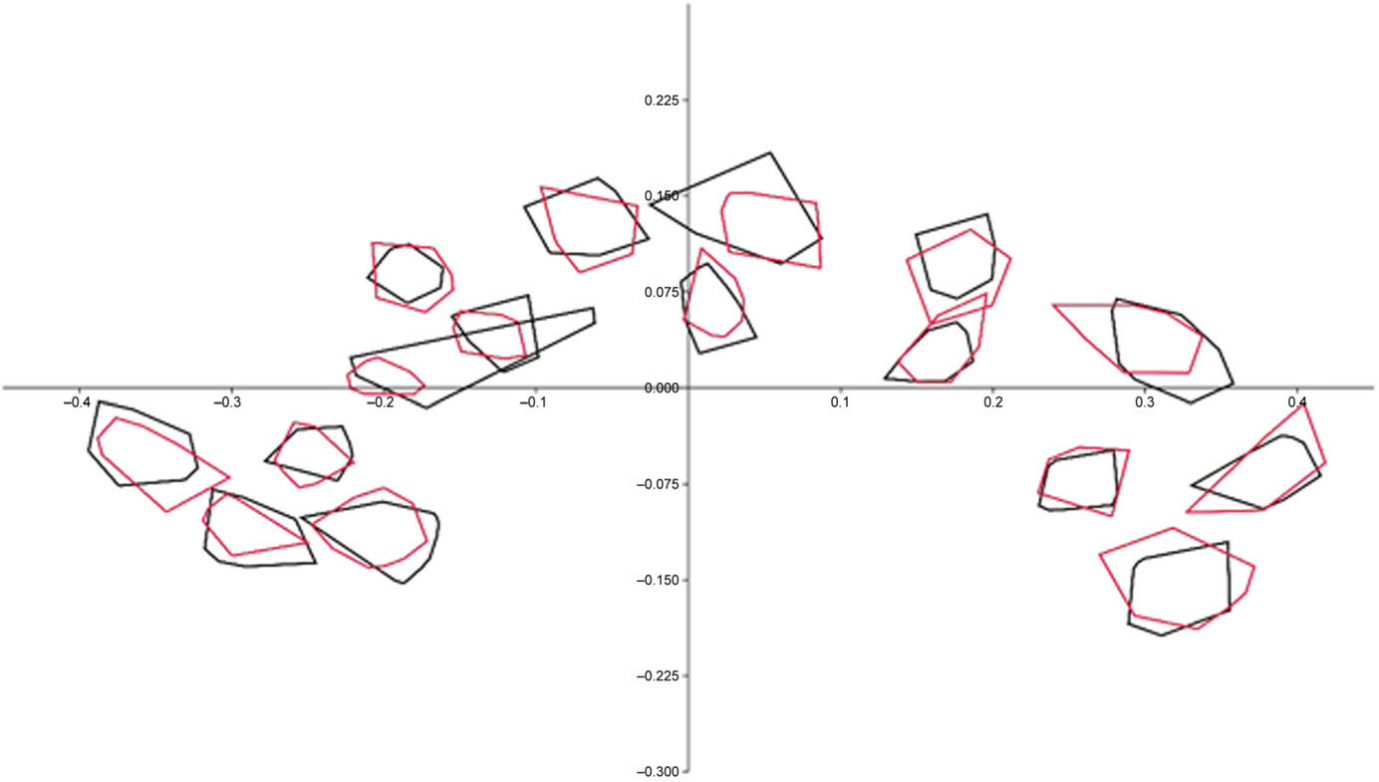
Ninety-five percent elliptic/convex hull graphs for landmark scatters in controls (grey) and bipolar disorder patients (red).

Since the Box-M test identified inhomogeneous matrices (F = 1.2152, *p* < 0.001), the James Fᴊ test was employed, and the findings demonstrated that CC shape was significantly different in BD patients when compared to the controls (T^2^ = 253.9534, *p* < 0.001) (Fig 2). The ratio of the root mean square determined by Kendall’s Riemann distance (rho) to the main shape was 0.08979253 in the control, 0.07942915 in the BD group, and 0.04313945 across all participants (Fig. 3).

**Figure 4.**
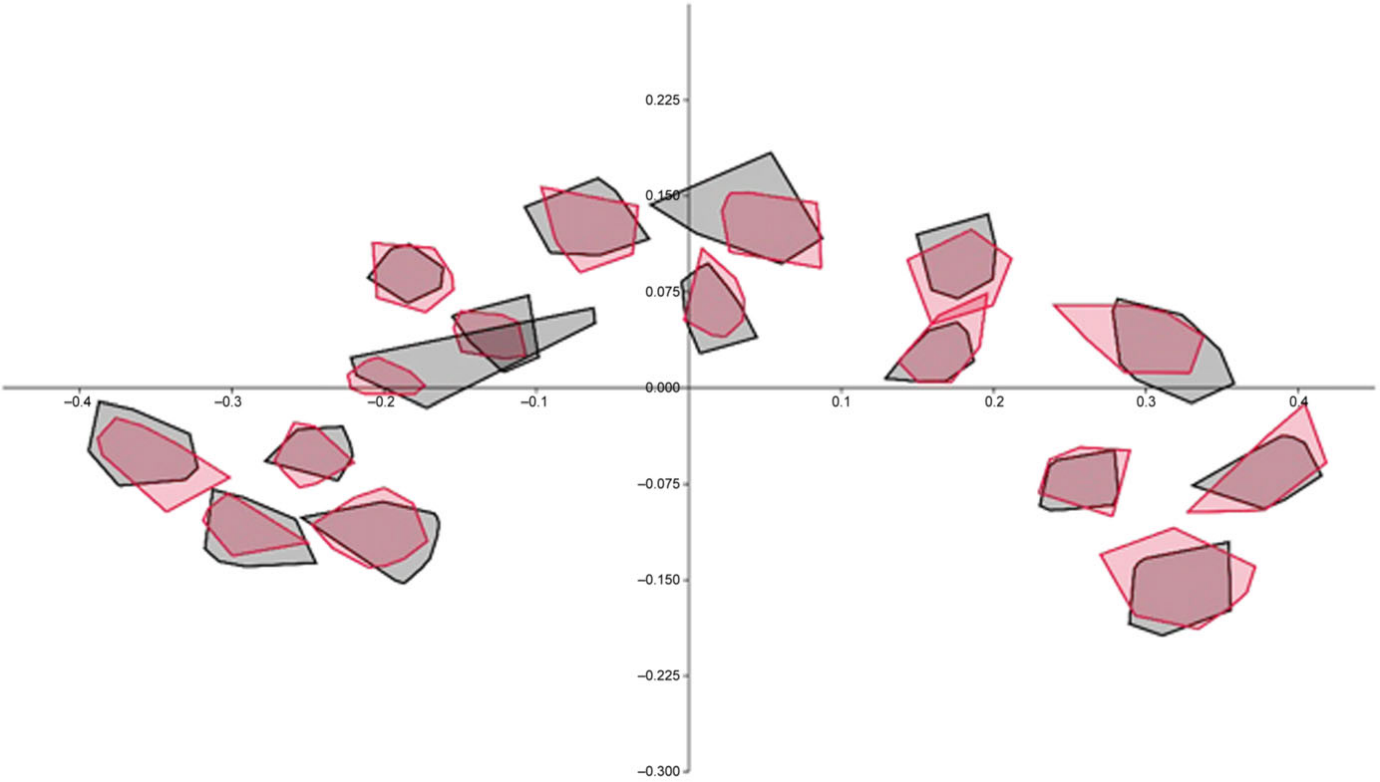
Thin-plate spline transformation grid with transformation expansion factors from the bipolar disorder group to the control group.

The impact of size-dependent shape variations and mean shape deformations observed on the graphs (shrinkage) was determined and compared between the control and BD patient groups with TPS (Fig. 4). Maximum deformation was observed at marked points (landmarks) in the anterior (genu) and posterior (splenium) CC regions (landmarks 5, 1, 3, 7, 4, 9, 12, 8, 6, 2, 10, 13, 11, 14, 16, and 15 in descending order) (Table 1).

**Table 1.** The mean dissimilarity and contribution of the landmarks

Landmark	Average dissimilarity	Contribution%	R/p
1	−8,90E + 11	13,02	−0.0115/<0.001
2	−1,35E + 13	4,10	
3	−4,14E + 12	12,72	
4	−3,23E + 13	7,53	
5	1,26E + 13	13,10	
6	−1,84E + 13	4,12	
7	−4,60E + 13	10,29	
8	−1,84E + 13	5,42	
9	−1,21E + 13	7,16	
10	1,68E + 13	3,75	
11	−7,11E + 12	3,04	
12	2,67E + 13	5,96	
13	−1,62E + 13	3,61	
14	−1,22E + 12	3,02	
15	6,48E + 12	1,45	
16	−7,65E + 12	1,71	

Multivariate regression analysis was employed to determine the correlation between size and shape in allometry analysis, and a statistically significant CC model (p = 5.84E-259, R^2^ = 0.13385, MSE = 0.0002093, and Wilks’ lambda = 1.798E–11) was obtained.

## Discussion

The current investigation found a substantial difference between BD patients and healthy controls in the analysis of SSA and CC in MRI images. The results of the investigation demonstrated aberrant white matter distribution and subregional abnormalities in BD patients’ CC. The two groups’ CC shapes differed significantly, with the posterior area markings showing the most CC deformation in BD patients. The CC area of BD patients and controls did not differ significantly.

The present literature that investigated the neurophysiology of BD with neuroimaging is limited. Nevertheless, the studies reported the likeliness of abnormalities in certain frontal-subcortical brain circuits. Future targeted studies are required to benefit from this novel technology to expand our knowledge of the neurophysiology of BD (Strakowski *et al*., 2000).

A study that analysed CC area and shape with two-dimensional shape analysis in first-stage psychotic schizophrenia, affective disorder, and healthy subjects reported no difference between CC areas in these three groups, while differences in shape were observed between schizophrenia patients and controls. Furthermore, the reduction of the CC width led to a decrease in the angle, resulting in a more curved shape in only the affective disorder group. These findings, while intriguing, should be verified with an independent group of subjects. Also, an intervention in the developing brain during the time of rapid CC growth could partially account for these findings, albeit speculative (Frumin *et al*., 2002).

BD and familial BD risk have been associated with abnormal WM microstructure in CC and front limbic pathways. These abnormalities could be trait or state markers, and it was suggested that these could be due to abnormal development and associated with difficulties in emotion regulation. Variations in the superior corona radiata/corticospinal tract and the CC body could represent a particularity marker of BD, while variations in other WM tracts could be a disease state marker. Finally, the relevance of WM microstructure in emotion regulation problems is a key feature of BD (Zhang *et al*., 2019).

Another study aimed to analyse the CC of patients with SSA and compare the findings with healthy controls. In the present study, SSA and CC analysis conducted on MRI images revealed significant differences between functional neurological disorder (FND) patients and healthy controls. The study findings emphasised abnormal WM distribution in CC and the variable subregional nature of the CC in FND cases (Baykara *et al*., 2022).

It could be hypothesised that abnormal maturation of brain connectivity could underlie dysfunctional emotion regulation in BD patients. To test this hypothesis, often WM integrity is investigated with water diffusivity measurements based on MRI. Here, a more intuitive aspect of the morphometry of the white matter tracts was considered: the shape of the fibre bundles, which is associated with neurodevelopment. Yi Sun Z, *et al*., analysed the shape of 3 tracts in BD: the cingulum (CG), uncinate fasciculus (UF), and arcuate fasciculus (AF). Their findings suggested neurodevelopmental abnormalities in the left AF in BD patients. The statistical trends observed in the left UF and CG suggested further research (Yi Sun *et al*., 2017). Certain studies that employed shape analysis reported significant findings on various CC subdivisions in schizophrenia (Joshi *et al*., 2013), autism subtypes (He *et al*., 2007), schizophrenia with deficit and non-deficit syndrome (Türk *et al*., 2021), mild cognitive impairment, and Alzheimer’s disease (Jiang *et al*., 2018). In Alzheimer’s disease, CC is globally atrophic, more prominently in the posterior segment, and the lower border is longer than the upper margin; in autism, there are differences in the CC body segment, and in multiple sclerosis, there are differences in the body and anterior segments of CC. The differences between the regional distribution differences in CC injury could be due to the variations in the regional fibre content in different neuroanatomical regions (Sigirli *et al*., 2012; Jiang *et al*., 2018).

Previous studies generally relied on volumetric and surface measurements, that is, quantitative features that can predict the progression of disease-induced changes. However, structural changes at specific locations are not adequately described in volumetric and superficial measurements. In the present study, maximum deformation was observed in the posterior (isthmus and splenium) and anterior (genu) regions in the CC. The first CC region, genu, connects prefrontal and cingulate areas with cognitive information (landmarks = 1, 7, 8, 9, and 12). The second area, splenium, connects temporal and occipital cortical areas. These are significant auditory, peripheral, and central visual stimulation regions (landmarks = 5, 3, and 4) (Walterfang *et al*., 2014; Friedrich *et al*., 2020; Fabri *et al*., 2013; Hofer and Frahm, 2006; Chao *et al*., 2009).

### Limitations

The present study has certain limitations. No scale was applied to the patients, and variables such as year of illness were not evaluated. Due to the lack of previous studies where CC was analysed with SSA in BD patients, we could not compare the study findings. The small patient and control sample size was another limitation of the current study. Although morphological analysis of the 3D data could seem difficult, it provides more accurate spatial morphological analysis results for organs. Also, the study participants were prescribed therapeutic mood stabilisers, antipsychotics, and antidepressants. To avoid affective episodes, BD cases were treated with mood stabilisers that included lithium, valproic acid, lamotrigine, and carbamazepine. Based on novel psychiatric approaches, the use of second-generation antipsychotics has also been considered in pharmacological treatment. There are preclinical studies where promitotic and antiapoptotic effects were induced by lithium. These studies supported the hypothesis of lithium-induced neurogenesis. However, the osmotic and physical effects of lithium could also explain the demonstrated volumetric gain in the bipolar human brain (Hamm *et al*., 2018).

## Conclusion

In the present study, SSA and CC analysis on MRI images revealed significant differences between BD patients and healthy controls. The study findings emphasised the abnormal white matter distribution and variations of subregional nature in CC in BD patients. Future studies with larger samples could contribute to further elucidation of these findings. The current study could assist future studies on the aetiology, diagnosis, and treatment options in BD.

## Data Availability

Not applicable.
